# Anti-*Helicobacter pylori* Effects of *Thymus caramanicus* Jalas Essential Oils: A New Antimicrobial Approach

**DOI:** 10.1155/2024/3627074

**Published:** 2024-06-10

**Authors:** Sepehr Asadi, Ebrahim Rahimi, Amir Shakerian

**Affiliations:** ^1^Department of Food Hygiene, Shahrekord Branch, Islamic Azad University, Shahrekord, Iran; ^2^Research Center of Nutrition and Organic Products, Shahrekord Branch, Islamic Azad University, Shahrekord, Iran

## Abstract

**Background:**

*Helicobacter pylori* are the principal causative factor in the etiological factors of chronic, active, or type B gastritis; peptic and duodenal ulcers; stomach carcinoma; and epithelial tissue lymphoid malignancies. It infects more than half of the population worldwide. To reduce *H. pylori* production, pharmacological therapy of *H. pylori* diseases typically involves using threefold treatment methods. However, as a result of such therapy, antimicrobial resistance is commonly developed. Alternative therapeutics for *H. pylori* diseases are thus of particular interest.

**Methods:**

Thyme essential oils (EOs) obtained from *T. caramanicus* Jalas plants in Iran were tested for antibacterial activity against *H. pylori* obtained from 320 poultry specimens in this investigation. Antibacterial activity was measured using inhibition zones, minimum inhibitory concentrations (MICs), and minimum bactericidal concentrations (MBCs). The impact of *T. caramanicus* Jalas essential oils on *H. pylori* isolate *cagA*, *vacA*, and *babA2* gene expression was evaluated using a quantitative real-time PCR method (*p* < 0.05).

**Results:**

The chemical content of these EOs varied significantly according to chromatographic examination. Thymol, carvacrol, and terpinene-4-ol are the most abundant components in these EOs. *H. pylori* was recognized as a *Helicobacter* species with a 175-bp PCR product of 16S rRNA in 20/20 (100%). According to PCR results, all 20 (100%) isolates belonged to *H. pylori*. The EOs inhibited *H. pylori* in a dose-dependent manner, with *T. caramanicus* Jalas being the most effective, followed by pterygium EOs in decreasing order. At 8 mg/mL of *T. caramanicus* Jalas EOs, IZs against *H. pylori* were 27.4 ± 0.42 mm, and at 8 mg/mL of pterygium, IZs against *H. pylori* were 1 ± 0.02. *T. caramanicus* Jalas essential oils were used to treat all bacteria, and the findings showed that *T. caramanicus* Jalas had a substantial inhibitory impact on the expression of *cagA*, *vacA*, and *babA2* virulence-related genes (*p* < 0.05).

**Conclusions:**

In a dose-dependent manner, the EOs of *T. caramanicus* Jalas EO demonstrated a high degree of antimicrobial property against *H. pylori* bacteria. The most efficient EOs were those from *T. caramanicus* Jalas with relative concentrations of thymol and carvacrol, followed by the coumarin-dominated pterygium EO with reduced antibacterial activity.

## 1. Introduction

*Helicobacter pylori* (*H. pylori*) is an underlying cause of the world's most common and chronic pathogenic bacteria, affecting about half of the country's population. Peptic ulcer disease, gastritis, stomach carcinoma, and functional dyspepsia are all believed to be caused by *H. pylori* [1–3]. Extradigestive diseases (idiopathic thrombocytopenic purpura, vitamin B12 insufficiency, and unexplained iron deficiency anemia) were also added to the criteria for *H. pylori* elimination [4, 5]. The prevention of *H. pylori* has proven difficult for clinicians recently due to the pathogen's developing antibiotic resistance. *H. pylori* diseases are generally treated with a proton pump inhibitor and two antimicrobials: amoxicillin, clarithromycin, or metronidazole (triple therapy) [6]. Levofloxacin can be used instead of clarithromycin during the first treatment, with greater efficacy [7]. Furthermore, an alternate experimental approach is required when regional clarithromycin tolerance exceeds 20% [8]. When the combination therapy fails, a bismuth-based quadruple medication (bismuth salts, tetracycline, and metronidazole with PPI) or a non-bismuth-based quadruple treatment (levofloxacin, nitazoxanide, and doxycycline plus PPI) should be advised [9]. Only symptomatic individuals require treatment. As a result, asymptomatic patients serve as a reservoir for *H. pylori* isolates in the community, particularly antibacterial drug strains that spread rapidly [10]. A dietary line focused on maintaining a low level of *H. pylori* concentration in the stomach mucosa would help people with microbial infections, limiting the establishment of acute gastroenteritis and an increased risk of gastric ulcers [11]. Urease is a crucial molecule that allows *H. pylori* to survive and colonize by starting the hydrolysis reaction, which produces ammonia, which is used to neutralize gastric acid and generate a favorable pH condition. As a result, urease is regarded as a crucial objective in developing and applying antimicrobial agents [12].

Vaccination, probiotics, photodynamic inactivation, phage treatment, and phytomedicine have all been researched as medicinal natural antimicrobial agents in past years [13]. The World Health Organization reports that various plant fractions and their dynamic constituents are utilized as traditional medicines by 80% of the world population. The medicinal potential of plant species is because of the occurrence of secondary phytoconstituents, which have numerous functions, such as antioxidant, antimicrobial, cytotoxic, anticancer, and antiviral [14, 15].

Numerous researches have been conducted in the quest for anti-*H. pylori* activities and gastroprotective effect in plants and plant herbal blends [16, 17]. Furthermore, natural-source components have been studied extensively as possible effective natural compounds for treating *H. pylori* infection. Antimicrobial, antifungal, antiviral, antiparasitic, and antioxidant activities have been discovered in herbal extracts [16]. Only a few studies have examined the impact of particular herbal extracts on *H. pylori* survival and proliferation [17]. The *in vitro* function of herbal extracts is poorly understood. The frequency of *H. pylori* in the gastrointestinal of mice treated with lemongrass was dramatically decreased compared to control animals, according to research by Ohno et al. [17]. Hartmani et al. evaluated the anti-*H. pylori* efficacy of a 2 : 1 blend of *Satureja hortensis* and *Origanum vulgare* subsp. hirtum aromatic plants. The combination efficiently eliminated this infection in 70% of the mice *in vivo* [18]. Plant-derived products have an imperative biological role against certain pathogenic organisms and were considered to be a major source of modern drugs [18].

*Thymus* (Lamiaceae) plants are well known as fragrant plants from the Eastern Mediterranean. In the Persian environment, 14 of the 215 species farmed globally are found [19]. *T. caramanicus* Jalas (Avishan) is an indigenous plant to Iran, and the leaves have historically been used for rheumatism, skin problems, and antimicrobial purposes [20]. In various regions of Iran, Thymus taxa are used as culinary herbs and teas. The antioxidant properties of pterygium and the composition and antioxidant capacity of the extracts of *T. caespititius*, *T. camphoratus*, and *T. mastichina* from Portugal have been documented [20–22]. Plant oils from various Thymus species, particularly *T. revolutus*, *T. pubescens*, *T. serpyllum*, *T. kotschyanus*, have been shown to have antibacterial action [23]. The plant oils from *T. caramanicus* were high in carvacrol at various developmental stages. The compounds also showed antimicrobial effects against Gram-positive and Gram-negative ATCC microbial species. Thymol has also been shown to have antimicrobial properties [24]. *Thymol* has been proposed as the physiologically active substance of thyme in the treatment of *H. pylori* [24, 25].

The objective of this study was to find EOs having potent antibacterial properties against *H. pylori* proliferation. *T. caramanicus* EOs were tested *in vitro* for anti-*H. pylori* activities. We investigated thyme EO's phytochemical profile and urease inhibitory capabilities with varied anti-*Helicobacter* activity.

## 2. Methods

### 2.1. Plant Identification and Collecting

Between July and September 2021, aerial portions of *T. caramanicus* Jalas were gathered. These wild plants were discovered in the research sites' common grounds. Furthermore, these taxa are not classified as threatened or protected. As a result, no special authorization was needed to collect and study the plants indicated. Voucher specimens had been kept in the Herbarium of I.A.U. Shahrekord Branch (no. 231).

### 2.2. Plant Material Preparation and Essential Oil Extraction

For the extraction of the essential oil in this study, the methodology of Chimnoi et al. [26] was used. Freshly harvested *T. caramanicus* Jalas plants (1.0 kg) were introduced to 2 L ethanol in a 4 L round-bottom glass beaker (with vegetal material/extraction solvent rate = 1/10 (w/v)) and exposed to water distillation for 3 hours using product obtained type apparatus. The essential oil was lighter than water and could be separated using a separating funnel. The oil was dried over anhydrous sodium sulfate. The amount of each oil was then measured in milliliters (mL), dried over anhydrous sodium sulfate, and kept in the dark at 4°C until use.

### 2.3. Analysis of Essential Oils

N-hexane was used to dilute 2 µL of *T. caramanicus* Jalas essential oils to 1 mL. A Shimadzu GC-2010 Plus gas chromatography system, connected to a Shimadzu QP2010 Ultra mass spectrometer, was used to evaluate 1 µL of essential oil liquids utilizing GC-MS (Shim-Pol, Izabelin, Poland).

A silicon dioxide capillary column ZB-5MS (30 m, 0.25 mm i.d.) with a layer thickness of 0.25 m was employed to isolate substances (Phenomenex, Torrance, CA, USA). The following oven temperature program was launched at 50 degrees Celsius, held for 3 minutes, then raised at 8 degrees Celsius each minute to 250 degrees Celsius and held for 2 minutes. The spectrum analyzers were set to electron-impact operation, with a wavelength of 40–500 amu, a 70-eV electron affinity, and a scan rate of 0.20 seconds per scanning. The pump, connector, and ion generator were all held at temperatures of 250, 250, and 220 degrees Celsius, respectively. Split infusion (1 *μ*L) was performed using a split frequency of 1 : 20 with helium as the gas phase at a flow rate of 1.0 mL/min. The stability indices were calculated using the same operation circumstances as a similar set of n-alkanes (C8–C20). A computer-assisted spectrum library (NIST 2011 Gaithersburg, MD, USA; MassFinder 2.1 and 4.0, Hamburg, Germany) used mass spectroscopy of reference compounds and MS information from the literature to identify substances.

### 2.4. Isolation and Identification of the *Helicobacter* Genus

Three hundred and twenty poultry samples were randomly gathered from farms in the Shahrekord region, Iran. The Gram-negative, S-, or C-shaped bacteria were seen by transferring the colonies on slants and staining them with gram. It was discovered that rod and coccoid forms exist. Biochemical tests were performed on the purified cultures to confirm their identity. As a control strain, the *H. pylori* ATCC 700392 strain was used. The *Helicobacter* genus was identified using 16S rRNA (Table 1). Lactofeed Biotech Group approved oligonucleotide sequences (Iran). Wilkins-Chalgren Anaerobe Broth-enhanced colonies were subcultured. Using a DNA extraction kit, genomic DNA was isolated from bacteria (Cinna-colon, Iran). The process was carried out according to the manufacturer's instructions. The extracted DNA's quality (A260/A280) and quantity were then tested (NanoDrop, Thermo Scientific, Waltham, MA, USA). The DNA's veracity was evaluated on a 2% agarose gel dyed with ethidium bromide (0.5 g/mL) (Thermo Fisher Scientific, St. Leon-Rot, Germany). A PCR thermal cycler (Eppendorf Co., Hamburg, Germany) was used to execute the polymerase chain reaction (PCR) according to the Piri-Gharaghie et al. protocol [23].

### 2.5. Screening for Antimicrobial Activity

#### 2.5.1. Determination of the Antibacterial Activities

The strains were produced during a 72-hour incubation period in an aseptic condition using brain heart infusion agar (BHA, Becton Dickinson, Germany) + 7% horse blood (5 percent O_2_, 15 percent CO_2_, and 80 percent N2). A densitometer (BioMerieux, Marcy l'Etoile, France) was used to calculate cell concentrations. The experiments were carried out using microbial solutions having a density of three on the McFarland scale or 3 × 108 cells (CFU)/1 mL. The European Committee on Antimicrobial Susceptibility Testing (EUCAST) (https://www.eucast.org) used Mueller-Hinton broth containing 5% lysed horse blood to evaluate essential oils for their antimicrobial properties through the microdilution broth technique.

#### 2.5.2. Determination of Minimum Inhibitory Concentration (MIC) and Minimum Bactericidal Concentration (MBC)

The investigated essential oils' minimal inhibitory concentration (MIC) was determined for all *H. pylori* isolates, with the procedure modified by adding resazurin after culture to see *H. pylori* development. On each microplate, there was a reasonable DMSO control (at a final concentration of 10%), a positive control (containing inoculum without the tested essential oils), and negative control (containing the tested essential oils without inoculum). Subculturing 5 *μ*L of the bacterial consortium from each well indicated growth inhibition from the final positive. The growth control on the prescribed agar plates yielded a minimal bactericidal concentration (MBC). The MBC was determined by the concentration of essential oil without microbial activity after 72 hours of incubation at 35°C in microaerophilic settings. Each investigation was carried out three times. The data are provided in a usual manner.

### 2.6. Antibacterial Virulence Gene Expression Analysis of EOs

A quantitative real-time PCR was used to evaluate the effect of the *T. caramanicus* Jalas essential oils on the *cagA*, *vacA*, and *babA2* gene expression of *H. pylori* isolate. Total RNA was extracted using a whole RNX-Plus kit (CinnaGen Co., Iran) from isolates after treatment with sub-MIC values of the *T. caramanicus* Jalas essential oils. The cDNA was synthesized according to the YTA Kit Protocol (Yekta Tajhiz, Iran). A real-time PCR test was performed with YTA SYBR Green master mix (Yekta Tajhiz, Iran), and the *16SrRNA* gene was used as an internal control. 15 *μ*L of reaction volume consisted of 0.5 *μ*L cDNA, 0.5 *μ*L forward primer, 0.5 *μ*L rivers primer, 10 *μ*L master mix, and 3.5 *μ*L of double sterile distillation water was used for real-time PCR. The temperature cycle program also included initial denaturation at 95°C for 10 minutes, followed by 40 cycles at 95°C for 20 seconds and 60°C for 40 seconds. The primer sequences of the target genes, including *cagA*, *vacA*, *babA2*, and *16S rRNA* (internal control), are given in Table 1.

### 2.7. Statistical Analysis

For each group, the data were reported as the mean + SEM. The statistical analysis was conducted using computer software (SPSS version 20). One-way analysis of variance (ANOVA) was used to compare the groups, followed by LSD post hoc multiple comparisons. *p* values of less than 0.05 were deemed statistically significant.

## 3. Results

### 3.1. Analysis of Essential Oils

**Figure 1:**
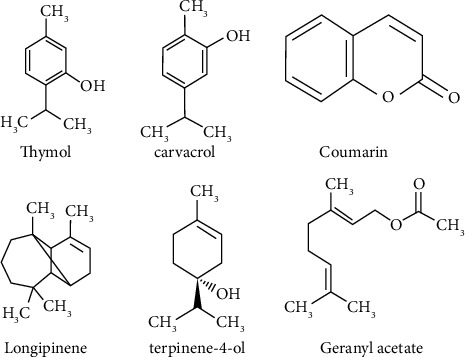
The structures of the key constituents found in the essential oils under investigation. Table 2's compound numbers are the same.

Table 2 lists the volatile components in *T. caramanicus* Jalas EOs in the elution frequency from a ZB-5MS phase. The chemical content of these EOs varied significantly according to chromatographic examination.  Figure 1 depicts the architecture of the most prominent terpenoids discovered in the studied EOs.

The monoterpene hydrocarbons are the primary distinguishing characteristics of the two EOs. *T. caramanicus* Jalas were used to make these EOs. However, the essential characteristics of T. caramanicus Jalas EO are the bicyclic monoterpenoids (3) and pinene (6). In contrast, the essential oil of pterygium is characterized by a monocyclic molecule, 2,2-bis(4-hydroxydiphenyl) propane. Monoterpene alcohols are the significant components in the two *T. caramanicus* Jalas chemically investigated EOs. This category includes essential oils hydrodistilled from *T. caramanicus* Jalas. Thymol, carvacrol, and terpinene-4-ol are the most abundant components in these EOs. Aside from alcohols, the existence of monoterpene hydrocarbons is a distinguishing feature of the EOs listed. p-Cymene is a critical component of thyme and oregano EOs, whereas additional terpinenes, such as *γ*, *α*, and *ε*-terpinene (=terpinolene), as well as -terpineol, were found in tea tree EO.

### 3.2. Isolation and Identification of the *Helicobacter*

In 320 cases of poultry flesh, the presence of *H. pylori* was evaluated. The 20 positive *Helicobacter* spp. were detected by urease, oxidase, and catalase assays after 4 hours of incubation, respectively, by a purple hue, a blue/purple color, and the generation of oxygen bubbles. *Helicobacter* spp. was found in 20 of 320 (6.25%) poultry meat specimens. The 16SrRNA gene PCR amplification was used to confirm all of the strains. The electrophoretically displayed PCR results from 20 Helicobacter spp. were identified from 320 poultry flesh specimens. *H. pylori* was recognized as a *Helicobacter* species with a 175-bp PCR product of 16S rRNA in 20/20 (100%). According to PCR results, all 20 (100%) isolates belonged to *H. pylori*.

### 3.3. Antibacterial Tests of *T. caramanicus* Jalas EOs against *H. pylori*

**Figure 2:**
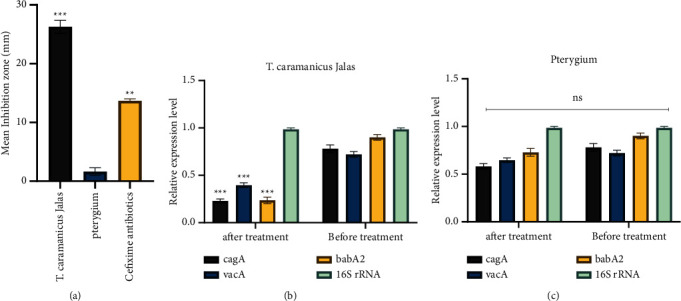
(a) Antibacterial tests of *T. caramanicus* Jalas EOs against *H. pylori.* (b) The effect of *T. caramanicus* Jalas on *H. pylori* virulence gene expression. (c) The effect of pterygium on *H. pylori* virulence gene expression. ^∗∗^*p* < 0.05 and ^∗∗∗^*p* < 0.05.

The EOs inhibited *H. pylori* in a dose-dependent manner, with *T. caramanicus* Jalas being the most effective, followed by *T. caramanicus* EOs in decreasing order (Figure 2(a)). The 8 mg/mL dosage of these *T. caramanicus* Jalas EO produced around 27.4 ± 0.42 mm inhibitory zones against *H. pylori*. The 8 mg/mL dosages, except pterygium EOs, resulted in less than 5 mm inhibitory zones (Table 3).

### 3.4. Results of Minimum Inhibitory Concentration (MIC) of Cefixime and EOs of *T. caramanicus* Jalas

In this test, the minimum inhibitory concentration of cefixime was compared to the performance of two extracts of *T. caramanicus* Jalas EOs. It is noted that *T. caramanicus* Jalas EOs have a high inhibitory concentration on *H. pylori* strains ranging from 16% to 72%.

The lowest number in each row of samples determined the best MIC performance. This is because the bacteria stopped growing at the same low dose of the drug. The more effectively bacterial growth is inhibited at a low concentration, the better candidate the essential oil is for treatment (Table 4).

### 3.5. Antibacterial Virulence Gene Expression Analysis of EOs

The virulence-related genes *cagA*, *vacA*, and *babA2* in MDR bacterial strains were assessed using a quantitative real-time PCR approach. *T. caramanicus* Jalas essential oils were used to treat all bacteria, and the findings showed that *T. caramanicus* Jalas had a substantial inhibitory impact on the expression of *cagA*, *vacA*, and *babA2* virulence-related genes (*p* < 0.05) (Figure 2(b)). The *T. caramanicus* Jalas significantly reduced the expression of *cagA*, *vacA*, and *babA2* genes in all MDR strains, which could be due to the interaction of thymol with transcription factors, which causes these factors to be inactivated, virulence genes to be not transcribed, and virulence gene expression to be reduced (Figure 2(c)).

## 4. Discussion

The plant's naturally extracted oils have been utilized in food storage, medicine, complementary therapy, and ecological cures for hundreds of years [27]. According to the previous research [28–30], natural compounds have antimicrobial effects on various bacteria. Moreover, aromatic plants differed in their antibacterial action. By measuring antimicrobial and antivirulence ability, we evaluated anti-*Helicobacter* characteristics of *T. caramanicus* Jalas essential oils commercially available. *T. caramanicus* Jalas (thyme) oil showed the highest activity against *H. pylori* strains. These EOs have significant biocompatibility against *H. pylori,* based on the biosynthetic pathway parameters of O'Donnell et al. [30]. The remaining EOs had good biocompatibility, with MICs ranging from 2 to 4%. Of the *T. caramanicus* Jalas EOs tested, the *T. caramanicus* Jalas essential oil had the lowest minimum inhibitory concentration (MIC).

Essential oils have previously been shown to have anti-*H. pylori* activity [31], and of the 13 plant extracts evaluated for their inhibitory activity on *H. pylori* in the study [32], the essential oils of *Cymbopogon citratus* (lemongrass) and *Lippia citriodora* (lemon verbena) were found to have the most potent antimicrobial activities for *H. pylori* strain ATCC 49503. Bergonzelli et al. (2003) studied the effects of 60 plant extracts on the development of *H. pylori in vitro* and found that 30 essential plant oils had a potent effect followed by 15 essential oils with a moderate effect. Carvacrol, isoeugenol, nerol, citral (=neral + geranium), and sabinene were found to have the strongest anti-*H. pylori* actions among the chemicals found in these oils [33]. Our research found monoterpenoids to be the most prevalent elements of the *T. caramanicus* Jalas EOs. This category of terpenoids accounts for over 90% of the total content of these herbal extracts. Sesquiterpenoids make up a minor portion of their overall makeup. Terpenoids are water-insoluble to relatively water-insoluble, although they dissolve in the phospholipid bilayer. Terpenoids are thought to have antibacterial properties because of their capacity to break or penetrate lipid structures, resulting in a loss of membrane stability, dissipation of the proton gradient, and disruption of internal pH equilibrium [34, 35]. The varying MIC values of the EOs may be related to their different phytochemical compositions. Monoterpene hydrocarbons were the most abundant components in pterygium, the least effective EO against *H. pylori* of the two EOs studied, implying marginal biocompatibility against *H. pylori*. Despite this minor variation, *T. caramanicus* Jalas had lower MIC values for the EOs than the pterygium group, probably due to the presence of monoterpene alcohols. The MIC values obtained increased as the concentration of EO increased. Thymol is a potent antibacterial agent, because of hydroxyl group and delocalized electrons essential for disrupting bacterial membranes [33], binding to transmembrane and intracellular molecules, and altering membrane fluidity, potassium ion permeability, and ATP contamination in microorganisms. Carvacrol and coumarin have a substantial inhibitory impact against *H. pylori* by disrupting and depolarizing the cell membranes by attacking membrane proteins and internal drug targets. However, the antimicrobial effect of carvacrol was diminished in the presence of thymol [36]. Thymol or carvacrol identified in *T. caramanicus* Jalas EO was discovered to be the primary components in our investigation, with the other two being identified in trace levels. Previous substances found in *T. caramanicus* Jalas EOs, such as -terpinene, _-longipinene, geranyl acetate, longifolene, (E)-*α*-carryophyllene, *α*-humulene, caryophyllene oxide, cedrol, and *α*-eudesmol, have been demonstrated to have no antimicrobial effect against Gram-negative bacteria in other research studies [32, 37, 38]. Moreover, certain substances with no antimicrobial activities have been observed to have a synergistic impact when combined with antimicrobial drugs; for instance, the existence of p-Cymene in combination with carvacrol may boost the oil's antibacterial effect [39]. The anti-*H. pylori* efficacy of the *T. caramanicus* Jalas EO is derived from monoterpene alcohols and aldehydes in pterygium EOs. Geranial and neral in *T. caramanicus* Jalas EOs have also been identified as compounds with increased anti-*H. pylori* antibacterial activities when compared to other materials in various research types [19].

A very different scenario existed in the instance of *T. caramanicus* Jalas EOs. There were no monoterpenoids found in this oil. Coumarin is the most prevalent in this EO, with cedrane being the most distinctive component. To date, no studies have been conducted on the biological action of pterygium EOs and their metabolites against *H. pylori*. Coumarin, which belongs to the pterygium family, has been shown to have anti-*H. pylori* action, as have the major components found in pterygium essential oils. Its antimicrobial property against *Streptococcus mutants* and antifungal characteristics has been described as dependent on DNA polymerase suppression [40]. Pterygium oil was not one of our research's most effective essential oils.

The generation of reactive oxygen and nitrogen molecules is linked to *H. pylori* infection preceding gastric mucosa colonization. As a result, antioxidants can be considered a supplemental medication for treating *H. pylori* [41]. Using nutritious nutritional supplements containing compounds with potent antioxidant activity can boost the body's defenses and prevent *H. pylori* from multiplying [42]. Furthermore, most research has shown that infectious disease reduces the number of antioxidants in stomach juice [43]. *In vivo* and *in vitro* research has revealed that antioxidants like vitamin C and astaxanthin scavenge not only oxidative stress but also have antibacterial action against *H. pylori* [44]. Bibi et al. (2017) earlier hypothesized a relationship between the existence of the *H. pylori babA2*/*cagA*+/*vacAs1* genotype and the prevalence of gastroenteritis, stomach carcinoma, and ulcerative colitis [45]. In *H. pylori* isolates recovered from clinical specimens of human and animal populations, a high incidence of *vacA*, *cagA*, *iceA1*, *oipA*, and *babA2* genotypes has also been described [45–47].

Moreover, *H. pylori* isolates recovered from varying dietary specimens have shown a significant frequency of these genes [48, 49]. Previous studies have linked the *H. pylori* genotypes *vacA*, *cagA*, *iceA*, *oipA*, and *babA2* to interleukin-8 and cytotoxin exudation, attachment to gastric epithelial cells, increase in the frequency of inflammatory impact, vacuolization, apoptosis process in gastric epithelial cells, stomach ulcers ulceration, and increased intense neutrophilic incursion [47–51]. In this study, the *T. caramanicus* Jalas significantly reduced the expression of *cagA*, *vacA*, and *babA2* genes in all MDR strains, which could be due to the interaction of thymol with transcription factors, which causes these factors to be inactivated, virulence genes to be not transcribed, and virulence gene expression to be reduced. The use of essential oils of *T. caramanicus* Jalas EO may aid in effectively managing *H. pylori*, which is widely disseminated worldwide. Furthermore, as the multiresistant profile grows, as does the public's desire to consume “green goods,” the usage of these EO will aid in managing *H. pylori* infection, perhaps reducing the pathogen's spread from asymptomatic carriers.

## 5. Conclusion

EO of *T. caramanicus* Jalas demonstrated a high degree of antimicrobial property in a dose-dependent manner against *H. pylori* strains. Its effectiveness may be due to its composition in bioactive molecules those with relative concentrations of thymol and carvacrol. However, *T. caramanicus* Jalas EOs showed a reduced antibacterial activity. In the end, the essential oil of *T. caramanicus* Jalas represents a better candidate for the formulation of an effective treatment against gastric ulcer caused by *H. pylori* strains.

## Figures and Tables

**Table 1:** List of primers used in this study.

Target gene	Oligonucleotide sequence (5′ -> 3′)	Size (bp)	Tm (°C)
16S rRNA	F: CTATGACGGGTATCCGGC R: ATTCCACCTACCTCTCCCA	175	53

VacA	F: AGCGCCATACCGCAAGAG R: CTGCTTGAATGCGCCAAAC	187	64

CagA	F: GATAACAGCCAAGCTTTTGAGG R: CTGCAAAAGATTGTTTGGCAGA	200	56

BabA2	F: CCAAACGAAACAAAAAGCGT R: GCTTGTGTAAAAGCCGTCGT	105–124	57

**Table 2:** Chemical composition of the analyzed essential oils.

No.	Compounds	Retention index on ZB-5MS column	Chemically analyzed essential oils	
			*T. caramanicus* Jalas	Pterygium
1	Thymol	1301	48.4	1.45
2	Carvacrol	1340	4.85	3.98
3	Terpinene-4-ol	1346	—	2.14
4	Coumarin	1351	—	50.2
5	_-Longipinene	1401	0.8	0.14
6	Geranyl acetate	1421	0.04	0.8
7	Longifolene	1428	2.6	3.6
8	(E)-*α*-caryophyllene	1489	2.4	3.8
9	*α*-humulene	1502	0.2	0.4
10	Caryophyllene oxide	1522	0.3	0.2
11	Cedrol	1529	0.2	—
12	*α*-eudesmol	1547	—	0.84

**Table 3:** Antibacterial activity of the *T. caramanicus* Jalas EOs using agar disk diffusion method.

*H. pylori*	Cefixime antibiotics (mm)	Essential oil extracts (8 mg/ml)	
		*T. caramanicus* Jalas	Pterygium
1	14 (I)	31 (S)	1 (R)
2	12 (R)	24 (S)	1 (R)
3	15 (I)	31 (S)	1 (R)
4	11 (R)	20 (S)	1 (R)
5	15 (I)	31 (S)	1 (R)
Mean ± SD	13.4 ± 0.2	27.4 ± 0.42	1 ± 0.02

**Table 4:** Minimum inhibitory concentration (MIC) and minimum bactericidal concentration (MBC) of *T. caramanicus* Jalas EOs.

*H. pylori*	Cefixime antibiotics (mg/ml)		*T. caramanicus* Jalas EO extract (8 mg/ml)		Pterygium EO extract (8 mg/ml)	
	MIC (mg/ml) (%)	MBC (mg/ml) (%)	MIC (mg/ml) (%)	MBC (mg/ml) (%)	MIC (mg/ml) (%)	MBC (mg/ml) (%)
1	8	16	2	4	16	32
2	8	16	2	4	36	72
3	8	16	4	8	72	144
4	4	8	2	4	16	32
5	8	16	4	8	36	72

## Data Availability

The data used to support the findings of this study are available from the corresponding author upon request.
